# The Association between Atherosclerotic Renal Artery Stenosis and Acute Kidney Injury in Patients Undergoing Cardiac Surgery

**DOI:** 10.1371/journal.pone.0064104

**Published:** 2013-05-21

**Authors:** Jingang Yang, Changlin Lu, Li Yan, Xinran Tang, Wei Li, Yuejin Yang, Dayi Hu

**Affiliations:** 1 State Key Laboratory of Cardiovascular Disease, Fuwai Hospital, National Center for Cardiovascular Diseases, Chinese Academy of Medical Sciences and Peking Union Medical College, Beijing, People's Republic of China; 2 Cardiovascular Center, Beijing Tongren Hospital, Capital Medical University, Beijing, People's Republic of China; 3 Department of Cardiology, Hebei General Hospital, Hebei Province, People's Republic of China; 4 Heart Center, People’s Hospital of Peking University, Beijing, People's Republic of China; Thomas Jefferson University, United States of America

## Abstract

**Background:**

Atherosclerotic renal artery stenosis (ARAS) and coronary artery disease (CAD) commonly co-exist. Some patients with unidentified ARAS may undergo cardiac surgery. While acute kidney injury (AKI) is a frequent and serious complication of cardiac surgery, we aim to evaluate the influence of ARAS on the occurrence of postoperative AKI in patients with normal or near-normal baseline renal function following cardiac surgery.

**Methods:**

A total of 212 consecutive patients undergoing aortography after coronary angiography and cardiac surgery were retrospectively studied for their preoperative and intraoperative conditions. AKI was defined as an absolute increase in serum creatinine of more than or equal to 0.3 mg/dl (≥26.4 µmol/l) or a percentage increase in creatinine of more than or equal to 50% (1.5-fold from baseline) after cardiac surgery. A propensity score-adjusted logistic regression models was used in estimating the effect of ARAS on the risk of postoperative AKI.

**Results:**

ARAS (≥50%) was observed in 50 (23.6%) patients, and 83 (39.2%) developed AKI after cardiac surgery. A correlation existed between renal artery patency and preoperative–to–postoperative %ΔCr in patients with ARAS (r = 0.297, P<0.0001). The propensity score-adjusted regression model showed the occurrence of postoperative AKI in patients with ARAS was significantly higher than those without ARAS (OR 2.858, 95% CI 1.260–6.480, P = 0.011).

**Conclusion:**

ARAS is associated with postoperative AKI in patients with normal or near-normal baseline renal function after cardiac surgery.

## Introduction

Acute kidney injury (AKI) is a frequent and serious complication of cardiac surgery. The incidence of AKI following cardiac surgery has been reported to vary between 1% and 30%, depending on the criteria used to define the complication. [1]–[3] AKI is an independent predictor for short- and long-term morbidity and in-hospital mortality, with a two fold to three fold increase in risk. [4] The etiology of AKI following cardiac surgery is poorly understood, but it is believed that ischemic injury of the kidneys, resulted from inadequate perfusion, is a major factor. In past several years, several investigators attempted to identify the risk factors for AKI after cardiac surgery. And peripheral vascular disease was found as one of the risk factors. [2], [5]–[13] Peripheral vascular disease and coronary artery disease commonly co-exist [14], [15] with incidental ARAS and atherosclerotic vascular disease elsewhere. Thus some patients with multiple coronary vessels disease may had unidentified ARAS when receiving coronary artery bypass graft (CABG) or valve replacement. There appear to be less data on the outcome of cardiac surgery in patients with renal artery stenosis as the cause of renal dysfunction. There was a case report that renal angioplasty prior to coronary surgery, in patients with concomitant renal and coronary artery disease, may reduce perioperative kidney injury, [16] while Conlon PJ et al. [17] showed renal artery stenosis was not associated with the development of acute renal failure following CABG. However they did find carotid artery bruit, a form of peripheral artery disease, was a risk factor of acute renal failure following CABG. Since atherothrombosis is a diffuse process, which suggested that patients with multiple-site atherosclerotic disease could predispose to perioperative renal dysfunction. We designed this study to evaluate the relationship between ARAS and AKI after cardiac surgery. Because duration of cardiopulmonary bypass (CPB) is associated with renal outcome, it has been proposed that avoidance of CPB with off-pump coronary bypass (OPCAB) may reduce perioperative renal insult. We also analyzed the effect of types of surgical procedures (CPB vs. OPCAB) on the postoperative renal function in patients with ARAS.

## Methods

### Patients

This was a retrospective cohort study performed at the cardiovascular center, Beijing Tongren Hospital, China. Data from a previously described cohort were used for the present study. [18] Among 859 consecutive patients undergoing abdominal aortography at the time of cardiac catheterization from March 2000 to October 2002, 212 patients were included in the study, which represented about one fourth of cardiac surgery performed in this period. Whether patients needed coronary angiography and CABG were decided by cardiologists who were not involved in the study. The results of screening abdominal aortography were communicated to the patients’ physicians. Patients with a serum creatinine level greater than 2.5 mg/dL (221 µmol/L) were excluded from consideration because of potential safety concerns about contrast volume administration. We also excluded infrequent procedures (ventricular assist device placement and adult congenital abnormality repair). This study was approved by the Ethics Committee of Cardiovascular Center, Beijing Tongren Hospital, Capital Medical University. Written informed consent was obtained from all participants.

### Procedure

Coronary angiography was performed with standard procedures. Arterial access was obtained with 6F intra-arterial sheaths using the modified Seldinger technique. After coronary angiogram, abdominal aortography was performed with a 6F pigtail catheter positioned above the level of the renal arteries to screen anatomical renal artery stenosis. Abdominal aortography was done by injecting 30 ml of Omnipaque 350 at a rate of 20 ml·s^−1^ to a total volume of 30 ml in a 10° left anterior oblique (LAO) projection, as the origins of the right and left renal arteries are well visualized in this projection. The injection was recorded at 30 frames per second. Selective renal artery injections were performed to detect distal renal artery stenosis, or when there were overlapping vessels were present.

For coronary lesions, significant stenosis was defined as a lumen narrowing ≥50%, in comparison with a proximal reference segment. Single-, double-, and triple-vessel disease were defined.

The aortographic image was independently interpreted by two experienced angiographers blinded to patient information. Significant renal artery diseases was defined as one or two renal arteries stenosis ≥50%. Severity of renal artery stenosis determined by aortograhy with a residual proximal renal artery patency scale. [19].

Cardiac surgical procedures were performed by the same team, which included CABG alone (n = 176) or combined aortic valve replacement (AVR; n = 8), mitral valve replacement (MVR, n = 6), AVR and MVR (n = 1), aortic root replacement (n = 1), AVR alone (n = 9); MVR alone (n = 11). Surgeries with CPB were in 151 patients and with OPCAB in 61 patients.

### Clinical Variables

Table 1 shows the demographic, clinical and laboratory variables. The demographic and anthropometric variables studied as potential risk factors were age, sex, height, weight, body mass index (BMI), and body surface area. The clinical variables evaluated were cardiomegaly (generalized cardiac enlargement on chest radiograph within 30 days of surgery), current tobacco use (within 2 weeks of surgery), diabetes mellitus (requiring therapy with oral agent or insulin), history of hypertension, left ventricular ejection fraction (assessed by two-dimensional echocardiography), NYHA functional class, percent stenosis of left main coronary artery, number of major coronary artery stenosis (≥50%), number of coronary artery anastomoses, preoperative use of an intra-aortic balloon pump (IABP) (within 1 week of surgery), prior history of myocardial infarction, time from contrast administration to surgery, systolic and diastolic blood pressure (mm Hg), and valve surgery. Current medication included digoxin, angiotensin-converting enzyme (ACE) inhibitors, nitroglycerin, beta-blockers, calcium channel blockers and diuretics (Table 2).

**Table 1:** Differences of baseline, intraoperative and postoperative variables between AKI and non-AKI patients.

	Non-AKI (n = 129)	AKI (n = 83)	P
Demography			
Age (y)	59.3±12.4	65.5±7.8	<0.0001
Male (%)	100 (77.5)	63 (75.9)	0.785
Weight (kg)	68.9±12.3	70.6±10.3	0.289
Height (m)	1.66±0.10	1.67±0.09	0.686
Body mass index (kg/m^2^)	25.0±4.8	25.4±3.1	0.475
Body surface (m^2^)	1.74±0.20	1.77±0.16	0.338
Preoperative parameters			
ARAS (%)	19 (14.7)	31 (37.3)	<0.001
Hypertension (%)	79 (61.2)	56 (67.5)	0.484
Diabetes (%)	38 (29.4)	31 (37.3)	0.232
Current smoker (%)	33 (25.6)	15 (18.1)	0.204
Stroke (%)	20 (15.5)	17 (20.5)	0.814
NYHA class	1.6±0.6	1.9±0.7	0.005
I (%)	60 (46.5)	25 (30.1)	
II (%)	57 (44.2)	43 (51.8)	
III (%)	12 (9.3)	13 (15.7)	
IV (%)	0 (0)	2 (2.4)	
Severity of CAD	2.3±1.1	2.7±0.8	0.009
0 (%)	20 (15.5)	4 (4.8)	
1 (%)	8 (6.2)	5 (6.0)	
2 (%)	16 (12.4)	7 (8.4)	
3 (%)	85 (65.9)	67 (80.7)	
Left main disease (%)	21 (16.2)	22 (26.5)	0.023
Ejection fraction (%)	60±12	58±12	0.268
Cardiomegaly (%)	37 (28.7)	20 (24.0)	0.254
Myocardial infarction (%)	43 (33.3)	32 (38.5)	0.438
Systolic pressure (mmHg)	120±17	123±15	0.293
Diastolic pressure (mmHg)	71±9	72±8	0.201
Time from contrast administration to surgery (day)	9.2±6.7	9.4±12.6	0.404
On IABP prior to surgery, %	4 (3.1)	6 (7.2)	0.179
Albumin (mg/L)	35.8±5.8	35.9±4.9	0.845
BUN (mmol/L)	6.6±2.7	6.6±2.1	0.532
CrPre (µmol/L)	90.6±22.1	92.9±26.0	0.490
Intraoperative parameters			
Valve surgery (%)	26 (20.2)	10 (12.0)	0.078
OPCAB (%)	40 (31.0)	21 (25.3)	0.371
Anastomoses	3.2±0.9	3.2±0.7	0.828
Postoperative parameters			
CrmaxPost (µmol/L)	97.7±23.5	169.9±84.0	<0.001
ΔCr (µmol/L)	21.2±20.5	130.5±80.0	<0.0001
%ΔCr	25±25	178±78	<0.0001

ARAS: atherosclerotic renal artery stenosis; IABP: intra-aortic balloon pump; BUN: blood urea nitrogen; CrPre: preoperative creatinine; NYHA: New York Heart Association; CAD: coronary artery disease; CrmaxPost: peak postoperative creatinine; ΔCr:difference between preoperative creatinine and postoperative creatinine; %ΔCr: percentage change of creatinine.

**Table 2:** Preoperative Medication Use in Patients Undergoing Cardiac Surgery.

	Non-AKI(n = 129)	AKI (n = 83)	P value
ACE inhibitors, %	91 (70.5)	61 (73.4)	0.148
Current diuretic, %	33 (25.6)	22 (26.5)	0.782
Nitroglycerin, %	72 (55.8)	54 (65.0)	0.182
Beta-blocker use, %	95 (73.6)	70 (84.3)	0.07
Digoxin, %	19 (14.7)	9 (10.8)	0.416
Lipid lowering agent, %	83 (64.3)	46 (55.4)	0.195
Calcium channel blocker, %	27 (20.9)	15 (18.1)	0.611

ACE: angiotensin-converting enzyme; AKI: acute kidney injury.

Serum creatinine was measured as routine laboratory tests for all cardiac surgery patients with a normal range of 0.7 to 1.3 mg/dL (62 to 124 µmol/L). The preoperative serum creatinine (CrPre) was the value on the day before surgery. Peak postoperative creatinine (Cr_max_Post) was defined as the highest value of during postoperative periods. Peak percentage change of creatinine (%ΔCr) was defined as the difference between the CrPre and Cr_max_Post. [20] AKI was defined as an absolute increase in serum creatinine of more than or equal to 0.3 mg/dl (≥26.4 µmol/l) or a percentage increase in creatinine of more than or equal to 50% (1.5-fold from baseline) after cardiac surgery [21].

### Statistical Analysis

Student’s t-test and chi-square or Fisher’s exact test were used, as appropriate, to evaluate the differences in continuous and categorical variables respectively. Continuous variables without an approximately normal distribution were analyzed by a nonparametric test (Wilcoxon’s rank sum). To test the effect of CPB on the development of AKI after cardiac surgery in patients with ARAS, we compared the %ΔCr in patients undergoing cardiac surgery with OPCAB vs. CPB. A correlation study was used to test the relationship between residual lumen patency, ΔCr and %ΔCr.

Propensity scores were the primary tool used to adjust the differences in the observed patient characteristics between groups. [22] In this study, propensity scores were calculated for each patient to estimate the probabilities of possible ARAS using multivariable logistic regression analyses. Confounding factors in logistic regression included age, sex, BMI, diabetes, hypertension, smoking, history of stroke and the severity of coronary artery disease. [19] Once formulated, the propensity scores was used as a single covariate in another multiple logistic regression model to compare the incidence rates of AKI between the patients with and without ARAS after adjusting for covariates (sex, age, BMI, preoperative serum creatinine, diabetes, hypertension, smoking, stroke, NYHA≥ Class II, CPB, IABP, the severity of coronary artery disease, left ventricular ejection fraction and systolic blood pressure). The level of statistical significance was assigned as P<0.05. The data were analyzed with SAS 9.3 software (SAS Institute, Cary, NC).

## Results

### Frequency and Severity of ARAS

All 212 patients were technically adequate for the evaluation of the renal artery anatomy. There were 50 (23.6%) patients with significant ARAS at the time of cardiac catheterization. Significant unilateral renal ARAS was identified in 33 (15.6%) patients, and 17 (8.0%) of them with significantly bilateral ARAS. Four patients had in essence one functional kidney due to total occlusion of one renal artery.

### Frequency and Severity of AKI

Postoperative AKI occurred in 83 (39.2%) patients, 62 (74.7%) of them on CPB and 21 (25.3%) on OPCAB. Among them, 2 (0.9%) patients required dialysis therapy. Serum creatinine values increased from 92.9±26.0 to 169.9±84.0 µmol/L in patients with AKI (P<0.0001). The AKI patients had an operative mortality at 7.2%, while the mortality in remainder of the cohort was 0.8% (P = 0.035).

### Predictors of AKI

The differences between AKI and non-AKI patients were presented in Table 1. AKI patients were older than non-AKI patients (65.5±7.8 vs. 59.3±12.4, P<0.0001). More severe cardiac function by NYHA classification was associated with the development of AKI (1.9±0.7 vs 1.6±0.6, P = 0.005). Patients with AKI showed a higher incidence of left main disease (26.5% vs. 16.2%, P = 0.023) or three-vessel coronary disease (80.7% vs. 65.9%, P = 0.009).

Time from cardiac catheterization and contrast administration to surgery was similar between groups. There were no significant differences in gender distribution, diabetes mellitus, hypertension, stroke, myocardial infarction and IABP use et al. Ejection fraction was measured in all patients by echocardiography before surgery, and the results were similar between groups. Preoperative medication use did not differ between AKI and non-AKI groups (Table 2).

The mean CPB duration was 95.3±41.3 min. No patient in OPCAB group converted to CPB. OPCAB did not significantly reduce postoperative AKI compared with cardiac surgery. The AKI occurrence rate was similar in patients who required valve replacement with or without concomitant CABG.

Patients with ARAS showed a significantly higher %ΔCr than those without ARAS (54.5±59.2 vs. 32.5±54.6, P = 0.001). Significant correlation between renal lumen patency and preoperative–to–postoperative %ΔCr was also observed (r = 0.297, P<0.0001). With propensity score-adjusted, AKI occurrence was significantly higher in patients with ARAS than those without ARAS (OR 2.858, 95% CI 1.260–6.480, P = 0.011) (table 3).

**Table 3:** Multiple Logistic Regression Adjusted by Propensity Scores.

	β	Wald χ^2^	P value	OR	95% CI
ARAS	0.525	6.3184	0.0119	2.858	1.260–6.480
Sex	0.2227	0.2235	0.319	1.561	0.650–3.749
Age	0.0581	0.0254	0.0224	1.06	1.008–1.114
Body mass index	0.0711	0.0443	0.0704	1.074	0.984–1.171
CrPre	−0.0083	0.0108	0.4425	0.992	0.971–1.013
Diabetes	−0.0667	0.1923	0.7287	0.875	0.412–1.860
Hypertension	0.1067	0.2316	0.645	1.238	0.499–3.068
Current smoker	0.2139	0.2022	0.2901	1.534	0.694–3.389
Stroke	−0.0669	0.2095	0.7495	0.875	0.385–1.988
≥NYHA class 2	−0.3365	0.248	0.1748	0.51	0.193–1.349
CPB	0.2918	0.3757	0.4373	0.558	0.128–2.433
IABP	0.1944	0.401	0.6278	0.678	0.141–3.264
Severity of CAD	0.4128	0.25	0.0987	0.438	0.164–1.167
Left main disease	0.3287	0.2338	0.1276	1.298	0.929–1.595
EF	0.0150	0.00666	0.6565	0.993	0.965–1.023
SBP	0.0214	0.0115	0.8529	0.998	0.976–1.021

ARAS: atherosclerotic renal artery stenosis; IABP: intra-aortic balloon pump; CAD: coronary artery disease; NYHA: New York Heart Association; CBP: Cardiopulmonary bypass; EF: ejection fraction; SBP: systolic blood pressure.

**Figure 1:**
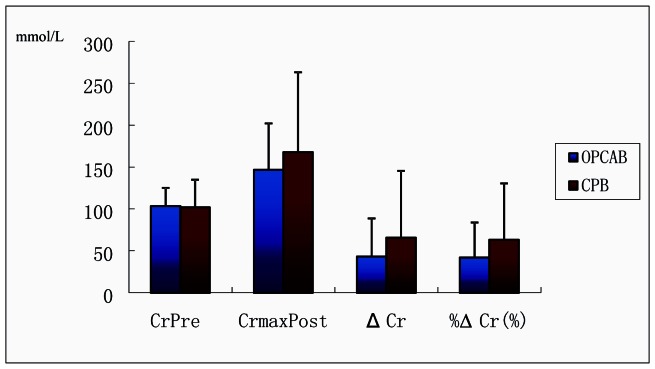
Changes of CrPre, Cr_max_Post, ΔCr and %ΔCr in Patients with ARAS and Undergoing Cardiac surgery. ARAS: atherosclerotic renal artery stenosis; OPCAB: cardiopulmonary bypass; OPCAB: off-pump coronary bypass; CrPre: CrPre: preoperative creatinine; CrmaxPost: peak postoperative creatinine; ΔCr: difference between preoperative creatinine and postoperative creatinine; %ΔCr: percentage change of creatinine.

However, the AKI occurrence rate was similar between patients with ARAS undergoing cardiac surgery with or without CPB. To further assess the effect of CPB and the extent of ARAS on the postoperative renal function in patients with ARAS, we compared ΔCr and %ΔCr in patients with ARAS undergoing cardiac surgery with (n = 30) or without CPB (n = 20). The values of ΔCr and %ΔCr in patients with OPCAB were a little lower than those with CPB, but not significantly (Figure 1).

## Discussion

Our study demonstrated that ARAS was associated with AKI after cardiac surgery, independent of the use of CPB. In addition, there is a correlation between renal artery patency and preoperative-to–postoperative %ΔCr in patients with ARAS. In previous studies, some authors identified that the history of peripheral vascular disease [2], [5]–[13], [23], carotid artery bruit [17], [23] or ascending aortic atherosclerosis [24] were the risk factors for AKI after cardiac surgery. These conditions could imply the severity of coronary disease and existing generalized atherosclerosis, while they were also related to the atherosclerotic ARAS. [18], [19], [25] Our results strongly support hypotheses that ARAS may also be a predictor of widespread atherosclerotic disease that may predispose to postoperative renal dysfunction.

A previous study, including 798 consecutive adult patients with a wide range of baseline serum creatinine undergoing CABG with CPB, presented that renal artery stenosis is not associated with the development of acute renal failure (serum creatinine increase ≥1 mg/dL) following CABG. [17] However, our result is different. First, we included the patients with valvular surgery because valvular surgery, combined CABG and valve procedure are risk factors for AKI after cardiac surgery. [26] Second, we excluded the patients with preoperative serum creatinine level ≥2.5 mg/dL, since abnormal preoperative serum creatinine level is one of the most important risk factors of AKI after cardiac surgery. [1], [2] Third, a robust multivariate logistic regression analysis using propensity scores was performed in our study.

The process causing renal dysfunction in patients with ARAS, peripheral vascular disease [2], [5]–[13], [23], carotid artery bruit [17], [23] or ascending aortic atherosclerosis [24] may be related to atheroembolism during surgical manipulation of an atherosclerotic aorta. The adverse effect of intraoperative atheroembolism during a cardiac operation has been long recognized. [27]–[31] Blauth and colleagues [31] showed that more than 10% patients who died after a cardiac operation had embolism in kidney. A study [24] showed a significant increase in the incidence of postoperative renal dysfunction as the severity of ascending aorta atherosclerosis increases from normal-mild (4. 1%) to moderate (9.0%) to severe (17.1%). The use of an intra-aortic filter captured 96.8% particulate emboli, and reduced postoperative renal complications for patients with moderate or greater preoperative risk [32].

Conlon PJ et al. have demonstrated that the duration of CPB is independently associated with the development of AKI and renal failure requiring dialysis. [23] With the revival of interest in performing CABG without CPB, OPCAB with complete avoidance of aortic manipulation may reduce the incidence of AKI. [33]–[35] However, there are different opinions. [36], [37] Our work also suggests that in patients with ARAS, off-pump surgery may not confer reduced risk of renal dysfunction compared with coronary bypass surgery with CPB. In this study, CPB did not seem to exert a deleterious effect on renal function in patients with ARAS. And of course, a randomized study with a larger sample size should be conducted to further elucidate this situation.

The process causing AKI in patients with ARAS in patients with ARAS may not solely due to atheroemboli. Chronic renal ischemia caused by ARAS elicits a complex biologic response. Besides of renin-angiotensin pathways underlying renal ischemia, there is evidence that additional mechanisms might be responsible for producing many of the hemodynamic alterations and end-organ injuries. [38] Renal injury in ARAS could be initiated by a reduction of nitric oxide (NO) bioavailability and increased intrarenal activity of the renin-angiotensin system, resulting in inflammation and the predominance growth promoting factors. [38], [39] Inflammation plays a central role in the development of ischemic kidney injury [40], [41]and it is thought that the systemic inflammatory response caused by cardiac surgery is similarly deleterious [4].

For the above reasons, although we have identified ARAS was associated with the development of AKI after cardiac surgery, we still do not know whether preventive revascularization can effectively prevent the occurrence of postoperative AKI. Moreover, some studies reported that stent implantation in patients with renal artery stenosis showed no beneficial effect on development of AKI after cardiac surgery. [42], [43] Since those kidneys typically exhibit nephron loss, nephrosclerosis, small vessel arteriosclerosis, and atheroembolic disease, lesions was not usually be reversible even with successful revascularization [44], [45].

There are several limitations for interpreting the present study. First, surgical selection bias was inherent in all but prospective, randomized trials. Further investigation is needed to establish the association between ARAS and post-operative AKI. Second, postoperative renal function was estimated with the change of serum creatinine. During postoperative period, however, these estimates may not be accurate because of imbalances between creatinine production and elimination, which could be caused by many factors, including changing renal function, muscle breakdown and injury, liver dysfunction, and various medications [46].

### Conclusion

Results from our study suggested that ARAS was associated with AKI after cardiac surgery and there is a correlation between renal artery patency and preoperative-to–postoperative %ΔCr in patients with ARAS. A large, prospective, randomized multicenter study is needed to provide an explicit explanation. We believe that ARAS screening is necessary when stratifying risk for the development of AKI, particularly in patients with multiple coronary artery lesions who planning to undergo CABG or other cardiac surgeries.

## References

[1] Huen SC, Parikh CR (2012) Predicting acute kidney injury after cardiac surgery: a systematic review. The Annals of thoracic surgery 93: 337–347. 22186469 10.1016/j.athoracsur.2011.09.010 PMC3286599

[2] Chertow GM, Lazarus JM, Christiansen CL, et al (1997) Preoperative renal risk stratification. Circulation 95: 878–884. 9054745 10.1161/01.cir.95.4.878

[3] Shaw A, Swaminathan M, Stafford-Smith M (2008) Cardiac surgery-associated acute kidney injury: putting together the pieces of the puzzle. Nephron 109: p55–60. 18802376 10.1159/000142937

[4] Rosner MH, Okusa MD (2006) Acute kidney injury associated with cardiac surgery. Clin J Am Soc Nephrol 1: 19–32. 17699187 10.2215/CJN.00240605

[5] Barbash IM, Ben-Dor I, Dvir D, et al (2011) Incidence and predictors of acute kidney injury after transcatheter aortic valve replacement. American heart journal 163: 1031–1036. 10.1016/j.ahj.2012.01.009 22709757

[6] Brandrup-Wognsen G, Haglid M, Karlsson T, Berggren H, Herlitz BJ (1995) Preoperative risk indicators of death at an early and late stage after coronary artery bypass grafting. The Thoracic and cardiovascular surgeon 43: 77–82. 7545332 10.1055/s-2007-1013775

[7] Rahmanian PB, Kwiecien G, Langebartels G, Madershahian N, Wittwer T, Wahlers T (2011) Logistic risk model predicting postoperative renal failure requiring dialysis in cardiac surgery patients. Eur J Cardiothorac Surg 40: 701–707. 21334918 10.1016/j.ejcts.2010.12.051

[8] D'Onofrio A, Cruz D, Bolgan I, et al (2011) RIFLE criteria for cardiac surgery-associated acute kidney injury: risk factors and outcomes. Congestive heart failure 16 (Suppl 1)S32–36. 10.1111/j.1751-7133.2010.00170.x 20653709

[9] Halkos ME, Chen EP, Sarin EL, et al (2009) Aortic valve replacement for aortic stenosis in patients with left ventricular dysfunction. The Annals of thoracic surgery 88: 746–751. 19699891 10.1016/j.athoracsur.2009.05.078

[10] Ahmadi H, Karimi A, Davoodi S, et al (2009) Determinant factors of renal failure after coronary artery bypass grafting with on-pump technique. Med Princ Pract 18: 300–304. 19494538 10.1159/000215728

[11] Brown JR, Cochran RP, Leavitt BJ, et al (2007) Multivariable prediction of renal insufficiency developing after cardiac surgery. Circulation 116: I139–143. 17846294 10.1161/CIRCULATIONAHA.106.677070

[12] Rodrigues AJ, Evora PR, Bassetto S, et al (2009) Risk factors for acute renal failure after heart surgery. Rev Bras Cir Cardiovasc 24: 441–446. 20305915 10.1590/s0102-76382009000500003

[13] van Straten AH, Hamad MA, van Zundert AA, Martens EJ, Schonberger JP, de Wolf AM (2010) Risk factors for deterioration of renal function after coronary artery bypass grafting. Eur J Cardiothorac Surg 37: 106–111. 19699103 10.1016/j.ejcts.2009.06.048

[14] Yang J, Hu D, Liu K, Li T, Peng J, Shang L (2002) High incidence of renal artery stenosis in patients undergoing coronary angiography. Zhonghua nei ke za zhi Chinese journal of internal medicine 41: 24–27. 11940292

[15] Yang JG, Li J, Lu C, Hasimu B, Yang Y, Hu D (2010) Chronic kidney disease, all-cause mortality and cardiovascular mortality among Chinese patients with established cardiovascular disease. Journal of atherosclerosis and thrombosis 17: 395–401. 20065612 10.5551/jat.3061

[16] Martin-Ucar AE, Patel RL (2002) Preventive stent placement for renal artery stenosis prior to emergent coronary artery bypass grafting. J Endovasc Ther 9: 218–220. 12010104 10.1177/152660280200900214

[17] Conlon PJ, Crowley J, Stack R, et al (2005) Renal artery stenosis is not associated with the development of acute renal failure following coronary artery bypass grafting. Renal failure 27: 81–86. 15717639

[18] Yang JG, Hu D, Li T, et al (2004) Angiotensin-converting enzyme inhibitor usage in patients with incidental atherosclerotic renal artery stenosis. Hypertens Res 27: 339–344. 15198481 10.1291/hypres.27.339

[19] Harding MB, Smith LR, Himmelstein SI, et al (1992) Renal artery stenosis: prevalence and associated risk factors in patients undergoing routine cardiac catheterization. J Am Soc Nephrol 2: 1608–1616. 1610982 10.1681/ASN.V2111608

[20] Ascione R, Nason G, Al-Ruzzeh S, Ko C, Ciulli F, Angelini GD (2001) Coronary revascularization with or without cardiopulmonary bypass in patients with preoperative nondialysis-dependent renal insufficiency. The Annals of thoracic surgery 72: 2020–2025. 11789787 10.1016/s0003-4975(01)03250-7

[21] Mehta RL, Kellum JA, Shah SV, et al (2007) Acute Kidney Injury Network: report of an initiative to improve outcomes in acute kidney injury. Critical care (London, England) 11: R31. 10.1186/cc5713 PMC2206446 17331245

[22] McCaffrey DF, Ridgeway G, Morral AR (2004) Propensity score estimation with boosted regression for evaluating causal effects in observational studies. Psychological methods 9: 403–425. 15598095 10.1037/1082-989X.9.4.403

[23] Conlon PJ, Stafford-Smith M, White WD, et al (1999) Acute renal failure following cardiac surgery. Nephrol Dial Transplant 14: 1158–1162. 10344355 10.1093/ndt/14.5.1158

[24] Davila-Roman VG, Kouchoukos NT, Schechtman KB, Barzilai B (1999) Atherosclerosis of the ascending aorta is a predictor of renal dysfunction after cardiac operations. The Journal of thoracic and cardiovascular surgery 117: 111–116. 9869764 10.1016/s0022-5223(99)70475-7

[25] Shurrab AE, Mamtora H, O’Donoghue DJ, al e (2001) Increasing the diagnostic yield of renal angiography for the diagnosis of atheromatous renovascular disease. Br J Radiol 74: 213–218. 11338095 10.1259/bjr.74.879.740213

[26] Thakar CV, Arrigain S, Worley S, Yared JP, Paganini EP (2005) A clinical score to predict acute renal failure after cardiac surgery. J Am Soc Nephrol 16: 162–168. 15563569 10.1681/ASN.2004040331

[27] Borioni R, Garofalo M, Pellegrino A, Actis Dato GM, Chiariello L (1994) Stroke prevention and carotid artery disease in cardiac surgical patients. The Annals of thoracic surgery 58: 1788–1789. 7979766 10.1016/0003-4975(94)91701-9

[28] Hosoda Y, Watanabe M, Hirooka Y, Ohse Y, Tanaka A, Watanabe T (1991) Significance of atherosclerotic changes of the ascending aorta during coronary bypass surgery with intraoperative detection by echography. The Journal of cardiovascular surgery 32: 301–306. 2055922

[29] Rokkas CK, Kouchoukos NT (1998) Surgical management of the severely atherosclerotic ascending aorta during cardiac operations. Seminars in thoracic and cardiovascular surgery 10: 240–246. 9801244 10.1016/s1043-0679(98)70024-3

[30] Wareing TH, Davila-Roman VG, Barzilai B, Murphy SF, Kouchoukos NT (1992) Management of the severely atherosclerotic ascending aorta during cardiac operations. A strategy for detection and treatment. The Journal of thoracic and cardiovascular surgery 103: 453–462. 1545544

[31] Blauth CI, Cosgrove DM, Webb BW, et al.. (1992) Atheroembolism from the ascending aorta. An emerging problem in cardiac surgery. The Journal of thoracic and cardiovascular surgery 103: 1104–1111; discussion 1111–1102. 1597974

[32] Banbury MK, Kouchoukos NT, Allen KB, et al.. (2003) Emboli capture using the Embol-X intraaortic filter in cardiac surgery: a multicentered randomized trial of 1,289 patients. The Annals of thoracic surgery 76: 508–515; discussion 515. 10.1016/s0003-4975(03)00530-7 12902095

[33] Kim KB, Kang CH, Chang WI, et al (2002) Off-pump coronary artery bypass with complete avoidance of aortic manipulation. The Annals of thoracic surgery 74: S1377–1382. 12400821 10.1016/s0003-4975(02)04060-2

[34] Massoudy P, Wagner S, Thielmann M, et al (2008) Coronary artery bypass surgery and acute kidney injury–impact of the off-pump technique. Nephrol Dial Transplant 23: 2853–2860. 18388121 10.1093/ndt/gfn153

[35] Seabra VF, Alobaidi S, Balk EM, Poon AH, Jaber BL (2010) Off-pump coronary artery bypass surgery and acute kidney injury: a meta-analysis of randomized controlled trials. Clin J Am Soc Nephrol 5: 1734–1744. 20671222 10.2215/CJN.02800310 PMC2974371

[36] Lamy A, Devereaux PJ, Prabhakaran D, et al (2012) Off-pump or on-pump coronary-artery bypass grafting at 30 days. The New England journal of medicine 366: 1489–1497. 22449296 10.1056/NEJMoa1200388

[37] Elmistekawy E, Chan V, Bourke ME, et al (2012) Off-pump coronary artery bypass grafting does not preserve renal function better than on-pump coronary artery bypass grafting: results of a case-matched study. The Journal of thoracic and cardiovascular surgery 143: 85–92. 22036259 10.1016/j.jtcvs.2011.09.035

[38] Murphy TP, Rundback JH, Cooper C, Kiernan MS (2002) Chronic renal ischemia: implications for cardiovascular disease risk. J Vasc Interv Radiol 13: 1187–1198. 12471181 10.1016/s1051-0443(07)61964-2

[39] Sigmon DH, Beierwaltes WH (1993) Renal nitric oxide and angiotensin II interaction in renovascular hypertension. Hypertension 22: 237–242. 8340159 10.1161/01.hyp.22.2.237

[40] Abuelo JG (2007) Normotensive ischemic acute renal failure. The New England journal of medicine 357: 797–805. 17715412 10.1056/NEJMra064398

[41] Sutton TA, Fisher CJ, Molitoris BA (2002) Microvascular endothelial injury and dysfunction during ischemic acute renal failure. Kidney international 62: 1539–1549. 12371954 10.1046/j.1523-1755.2002.00631.x

[42] Erentug V, Bozbuga N, Polat A, et al (2005) Coronary bypass procedures in patients with renal artery stenosis. Journal of cardiac surgery 20: 345–349. 15985135 10.1111/j.1540-8191.2005.200444.x

[43] Zheng B, Yan HB, Liu RF, et al (2011) Is it necessary to stent renal artery stenosis patients before cardiopulmonary bypass procedures? Chinese medical journal 124: 1453–1457. 21740797

[44] Koivuviita N, Liukko K, Kudomi N, et al (2012) The effect of revascularization of renal artery stenosis on renal perfusion in patients with atherosclerotic renovascular disease. Nephrol Dial Transplant 27: 3843–3848. 22785108 10.1093/ndt/gfs301

[45] Wheatley K, Ives N, Gray R, et al (2009) Revascularization versus medical therapy for renal-artery stenosis. The New England journal of medicine 361: 1953–1962. 19907042 10.1056/NEJMoa0905368

[46] Bloor GK, Welsh KR, Goodall S, Shah MV (1996) Comparison of predicted with measured creatinine clearance in cardiac surgical patients. Journal of cardiothoracic and vascular anesthesia 10: 899–902. 8969398 10.1016/s1053-0770(96)80053-x

